# Multiscale fractal dimension applied to tactical analysis in football: A novel approach to evaluate the shapes of team organization on the pitch

**DOI:** 10.1371/journal.pone.0256771

**Published:** 2021-09-01

**Authors:** Murilo José de Oliveira Bueno, Maisa Silva, Sergio Augusto Cunha, Ricardo da Silva Torres, Felipe Arruda Moura

**Affiliations:** 1 Sport Sciences Department, State University of Londrina, Londrina, Brazil; 2 Institute of Computing, University of Campinas, Campinas, Brazil; 3 College of Physical Education, University of Campinas, Campinas, Brazil; 4 Department of ICT and Natural Sciences, NTNU—Norwegian University of Science and Technology, Ålesund, Norway; Instituto Politecnico de Viana do Castelo, PORTUGAL

## Abstract

The aim of this study was to evaluate different shape descriptors applied to images of polygons that represent the organization of football teams on the pitch. The effectiveness of different shape descriptors (area/perimeter, fractal area, circularity, maximum fractal, rectangularity, multiscale fractal curve—MFC), and the concatenation of all shape descriptors (except MFC), denominated Alldescriptors (AllD)) was evaluated and applied to polygons corresponding to the shapes represented by the convex hull obtained from players’ 2D coordinates. A content-based image retrieval system (CBIR) was applied for 25 users (mean age of 31.9 ± 8.4 years) to evaluate the relevant images. Measures of effectiveness were used to evaluate the shape descriptors (P@n and R@n). The MFD (P@5, 0.46±0.37 and P@10, 0.40±0.31, p < 0.001; R@5, 0.14±0.13 and R@10, 0.24±0.19, p < 0.001) and AllD (P@5 = 0.43±0.36 and P@10 = 0.39±0.32, p < 0.001; R@5 = 0.13±0.11 and R@10 = 0.24±0.20, p < 0.001) descriptors presented higher values of effectiveness. As a practical demonstration, the best evaluated shape descriptor (MFC) was applied for tactical analysis of an official match. K-means clustering technique was applied, and different shapes of organization could be identified throughout the match. The MFC was the most effective shape descriptor in relation to all others, making it possible to apply this descriptor in the analysis of professional football matches.

## Introduction

With the possibility of obtaining data on the athletes’ position as function of time, analysing the tactical formation of players of teammates and opponents, as well as patterns of play, has been the aim of recent research on football [1–5]. Several computational methods have been developed and applied on the last decade to understand tactical behavior in team sports, related to spatial parameters [6–8]. These variables are presented in the literature as relevant components for the analysis of the organization of football teams on the pitch, such as centroid, spread, stretch index, width, length and the surface area that the teams cover on the pitch [3, 9–11]. In the literature, the surface area is usually represented by the area of a polygon, obtained from the position of the players of the same team. Which represents the total space covered by a given team on the pitch [3, 12].

Although the absolute values of the surface area represent good parameters for the characterization of teams’ organization during a match and are largely used [1, 3, 9, 13], it can be argued that different distributions of players on the pitch can provide equal surface areas or, for similar organization shapes, it is possible that the teams present different areas, therefore, only by magnitude of the surface area (m^2^), it is not possible to establish how teams are organized. In this sense, a more detailed analysis of the structural shape of the polygon, represented by the surface area of football teams on the pitch, can provide information on the complexity of organization during office matches. In fact, the way teams act on the pitch is associated with their organizations to achieve success in the modality.

The structural shape of an object is considered an important feature to describe and distinguish certain objects [14]. Some shape descriptors are presented in the literature for the characterization of a given object, such as the circularity, rectangularity, and length of the major and minor axes of images (main axes) [15]. These descriptors are basically classified into two categories: analysis based on contour or based on region [16]. As the labels suggest, the first uses only information from the object’s outline, while the second uses all object pixels to obtain a vector of characteristics.

An effective approach to describe and to estimate the complexity of a given shape relies on the use of measures based on fractal dimension. The fractal geometry [17] is a field of mathematics often employed for analysing the complexity of a shape. This theory has been used in image processing, specifically for texture segmentation [18] and image compression [19]. One of its fundamental definitions of the fractal dimension is a measure of complexity that generalizes the concept of topological dimension. The Multiscale Fractal Dimension (MFD) is an extension of the fractal dimension *Minkowski-Bouligand*, which uses Euclidean morphological dilation for multiscale representation, as characteristic of self-similarity [20].

Once tools that describe the properties of polygons’ shapes associated with the organization of football teams are not presented in the literature, it is necessary to implement, evaluate, and validate methods of descriptors in ways that can better describe these polygons. Therefore, the aim of this study was to evaluate different shape descriptors applied to images of polygons that represent the organization of football teams on the pitch and to establish which is the best descriptor(s) to describe the shapes of the team organization throughout a match. The initial hypothesis is that the MFD may present better results to describe the shapes of the organization of football teams, according to the fact that the MFD is not invariant at scale [20], which allows the identification of shapes with similar complexity considering the respective scale (different areas for example). Additionally, we demonstrated a possible application of the best evaluated descriptor to describe the organization of football teams throughout an official match.

## Materials and methods

### Data collection

To obtain the surface area and obtain the polygons corresponding to the shape of organization of the teams on the pitch as a function of time of a match, the 2D position of the players on the pitch was obtained using the DVideo software [21, 22]. The software has an average error of 0.3 m for determining the player’s position and an average error of 1.4% for the distance covered by the players [22, 23]. The study protocol was approved and followed the guidelines stated by the local Institution–Ethics Committee of State University of Londrina (3.047.461)–and in conformity with the recommendations of the Declaration of Helsinki.

### Elaboration of the polygons that represent the tactical organization of the football teams

**Fig 1:**
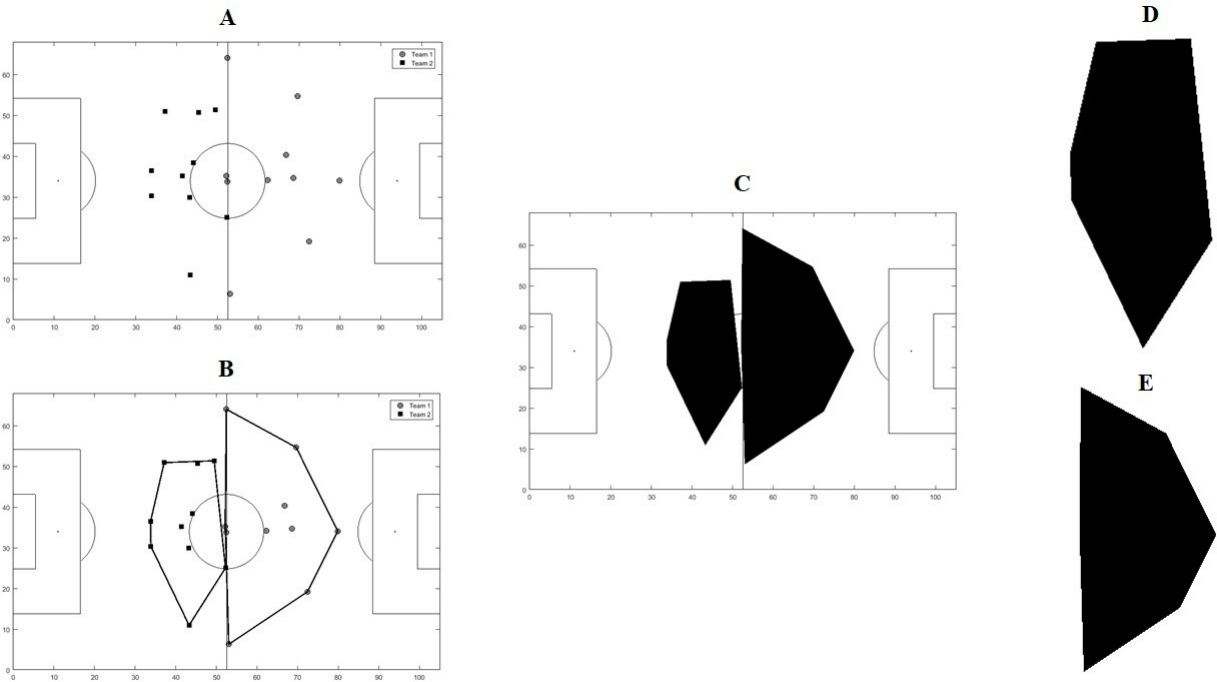
Steps for the construction of images of the convex polygons that represent the organization of the teams in the field: (A) Position of the players of each team at a given time; (B) Application of the quickhull technique for the construction of the convex hull; (C) Convex hull polygon in the respective field position; Images of the shapes analysed for each team, (D) Team 1 and (E) Team 2.

As the object of study to be analysed, images of polygons were generated in MATLAB® environment for an official match of the Brazilian first division football championship of both teams involved. To represent the organization of teams in the pitch as a function of time (*t*), the convex hull was generated [3, 24]. The convex hull of a set of points S on a plane (in this case, represented by players’ position on in pitch, without the goalkeeper (Fig 1A), at each instant of time t, is the smallest convex set that contains S (Fig 1B and 1C). If S is finite, the convex hull is always a polygon whose vertices are a subset of points S. The convex hull was computed by the QuickHull technique [24]. Finally, for each team, binary images of the shapes of the convex hull (Fig 1D and 1E) were stored at each instant of time.

### Calculation of shape descriptors

For this study, different shape descriptors were calculated for the analyses of which ones can better represent the images of the analysed polygons, as described below:

#### Multiscale Fractal Dimension (MFD)

The fractal dimension (FD) is largely defined to characterize objects in terms of complexity through fractional values and their self-similarity [25, 26]. Among these definitions, the Minkowski-Bouligand fractal dimension has been one of the most popular in image analysis [20]. This method allows the description of objects as a function of different dilations (scales), obtaining a greater number of information (vectors of characteristics) that can distinguish objects in different scales, being the multiscale fractal dimension (MFD) [20, 27]. The MFD is calculated according to (Eq (1)):

$$MFD=2-\underset{r\to 0}{\lim}\frac{\log\left(A(r)\right)}{\log\left(r\right)}$$ (1)

where *A(r)* is the area of a region expanded by a radius *r*. The algorithm consists of three steps, reported below, used to calculate the Minkowski-Bouligand fractal dimension [20].

**Fig 2:**
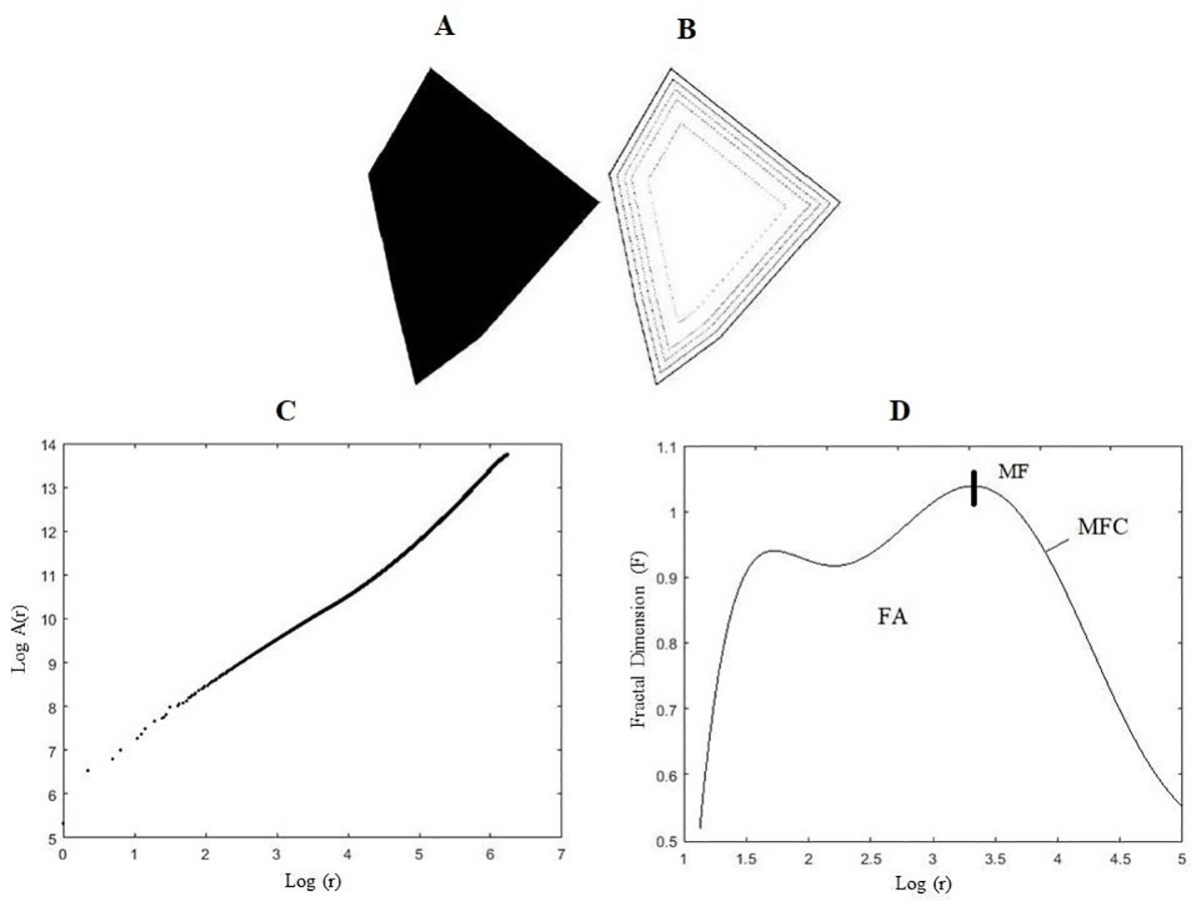
(A) Image of a polygon; (B) dilated contours; (C) Logarithmic function of the areas of exact dilation radius; (D) Multiscale Fractal Dimension of the analysed image, with Maximum Fractal Value (MF), Multiscale Fractal Curve (MFC), Fractal area (FA).

- **Euclidean Distance Transformation (EDT) by Image-Foresting Transform:** this step consists of the execution of the Image-Foresting Transform (represented in this case by the cost map) to perform the EDT of the images. Given a set of points S in a Cartesian plane (x, y), the expansion occurs by propagating the cost to adjacent pixels according to the Euclidean distance matrix, provided that the new cost of the adjacent pixel is less than the previous cost. This is a way to obtain simplified progressive values of the format (Fig 2A and 2B). The greater the distance values are, the greater the simplicity of the shape and, consequently, the smaller the details and similarities.
- **Evaluation of areas of dilated contours:** each multiscale instance S(*r*) of the original form S is obtained by the threshold of the cost map in a given Euclidean distance *r* squared; in this way, it is possible to obtain the area of a multiscale instance, A(*r*), through the cumulative histogram of the cost map. From Eq 1, it is also necessary to compute the log x log of these cumulative histograms.
- **Estimate of the multiscale fractal dimension:** defined as a common approach to evaluate a fractal dimension with a single linearly adjustable value, the *logA(r)* x *log(r)* curve, and considers fractal dimension F to be 2 minus the slope. For this study, a previously proposed method was used [20], whose curve with a polynomial $f_{n}^{\prime }(r)$ with n degrees is adjusted, by means of regression, to the logarithmic function of the area from which the derivatives can be obtained immediately. Thus, the fractal dimension of the dilated contours is obtained as a function of the dilation radii, (Eq (2)):

$$F(r)=2-f_{n}^{\prime }(r)$$ (2)

Fig 2C presents the result of the log x log cumulative histogram of the filtered cost map with a polynomial of degree n (n = 10) for a vector of characteristics with a sample of 100 points in an example using the contour shown in (Fig 2B). Finally, (Fig 2D) presents the final result of the MFD for (Fig 2A).

After all the steps described above were performed, multiscale fractal curve (MFC) was obtained for each image in the database. The MFC consists of the values of the scalar fractal dimension, a variant of a given polygon. To characterize the shape of the figures, the maximum value of the MFC (MF) was also identified, and the integral of the MFC was calculated, obtaining the fractal area (FA) values below the MFC.

#### Rectangularity

The rectangularity can be defined as a measure of how well a shape can approach the smallest rectangle that delimits the polygon (MER). The rectangularity is calculated according to (Eq (3)):

$$Rectangularity=\frac{A(shape)}{A(MER)}$$ (3)

where *A(shape)* represents the area of the polygon (pixels) and *A(MER)* represents the area of the smallest rectangle that delimits the polygon (square pixels).

#### Circularity

In this study, the circularity was defined according to (Eq (4)):

$$Circularity=\frac{P^{2}}{A}$$ (4)

where *P* and *A* denote the perimeter and area, respectively. Both measures (rectangularity and circularity) are dimensionless [15].

#### Area/Perimeter ratio

The shape descriptor that demonstrates the Area/Perimeter ratio (AP) was also calculated according to (Eq (5)):

$$AP=\frac{A}{P}$$ (5)

where *A* and *P* denote the area and perimeter of the polygon, respectively.

#### Alldescriptors

Finally, a feature vector (FV) was created by concatenating the values of all descriptors, denominated Alldescriptors (AllD). In this FV, data from the MFC were not included. It was organized as follows:

Alldescriptors = [AP FA Circularity MF Rectangularity];

With possession of the shape descriptor values calculated for each polygon as a function of time, a system was implemented to evaluate the descriptors and identify the most relevant polygons in relation to the query images randomly selected in the same collection images. Therefore, a Content-Based Image Retrieved (CBIR) system was used for the evaluation of descriptors [28], described in the following section.

### Content-Based Image Retrieved (CBIR) system: Steps for implementation

For the implementation of a CBIR system, the image descriptor is very important, as it is used to assess the similarity between the query and collection images [28]. In this study, 25 queries were selected randomly from the same collection images. These queries represent different polygons that can be extracted from the same image base, which makes it possible to explore the wide variety of shapes a team can present throughout a match. The process for preparing a CBIR occurs in the following order [29]:

- The shape descriptors for queries were calculated, as well as for all collection images. This step is important for the content-based image retrieval. Subsequently, a similarity analysis was performed between the query and the collection images.
- The similarity analyses between the query and the collection images were computed by means of the Euclidean distance (in computation denominated L2) between the values of the FV of each query for all collection images.
- A ranking of the collection images was performed based on the Euclidean distance, from the most similar (least distant) to the least similar (most distant) to the queries. This step was performed so that the CBIR allowed us to identify the most relevant images of each descriptor for each query. One descriptor is considered better than another if, when used, it leads to more relevant results in the first positions of the ranking.

After accomplishing all the steps to build a CBIR, the relevant images (i.e. similar images) obtained by CBIR were evaluated by real users, according to the guidelines of [29, 30]. For each analysed descriptor, the first 12 most relevant images of each query were retrieved. For each list of images selected in each model, these images were combined into a list, and then duplicates were removed. In the end, the image lists were shuffled before being displayed to users through an interface created specifically for this study.

### Evaluation of the effectiveness of shape descriptors

#### Participants

For this study, 25 users (students and professionals of Sports Sciences) of both genders (31.9 ± 8.4 years old), were invited to evaluate relevant images obtained by CBIR. Users visited the laboratory in a single session and signed an informed consent form. The study protocol was approved and followed the guidelines stated by the local Institution–Ethics Committee of State University of Londrina (3.047.461)–and in conformity with the recommendations of the Declaration of Helsinki.

#### Interface to evaluating relevant images

An interface was created in the MATLAB^®^ environment, in which a given query was highlighted to the participants, with images to be analysed. The user, using the interface, analysed 84 possibly relevant images for each three queries, with a total of 252 images. These images were presented on pages of six available images for the same query and the user identified which of the images presented were most relevant in relation to the query. After identifying the relevant image(s), we instructed the users to select the image considered relevant. This process was performed for the three queries evaluated, separately. As soon as each participant ended the evaluation, a data matrix was generated and saved with the information of which images the participant considered relevant for each query.

#### CBIR evaluation

Precision and recall measures were calculated to assess the effectiveness of the ranked lists produced by different descriptors. The precision measures the fraction of relevant images returned by the user in a given query (an input polygon) in relation to the total number of images. The recall measures the fraction of relevant images returned for a given query in relation to the total of relevant images existing in the collection images [29].

The precision for nth position (P@n) and the recall for nth position (R@n) were calculated for a given number of images, which provides an assessment of the user’s impression of the ranked results. P@5 and P@10 provide reliable metrics to assess whether the search obtains relevant images at the top of the ranking. Thus, the higher the concentration of relevant images at the top of the ranking, the more effective the descriptor is considered [31].

The evaluation of the effectiveness of the system was measured using a precision x recall curve and P@5, P@10 (Eq (6)), R@5 and R@10 (Eq (7)) values. Below are presented the calculations:

$$Precision=\frac{r}{n}$$ (6)

$$Recall=\frac{r}{rt}$$ (7)

where *r* is the number of relevant images to the descriptor, n is the total number of images in the collection and *rt* is the total number of images considered relevant for each query.

### Statistical analysis

A measure of interpersonal agreement was tested by calculating the Kappa coefficient [32] to verify the agreement between different users in identifying the relevant images for the queries. The concordance analysis was performed for each of the 25 queries. Each query was evaluated for three individuals namely A, B, and C. Thus, all subjects participated in the concordance analysis performing one evaluation, resulting in three evaluations for each of the 25 queries. The concordance analyses were performed in pairs (A x B; A x C; and B x C). The following reference values were considered: k ≤ 0.2 Slight agreement; k > 0.2–0.4 Fair agreement; k > 0.4–0.6 Moderate agreement; k > 0.6–0.8 Substantial agreement; k > 0.8 Almost perfect agreement [33].

Levene’s test was performed to analyse the homoscedasticity of the data. A sphericity analysis was applied using Mauchly’s test. One-way analysis of variance of repeated measures was applied to verify whether there were differences between the descriptors for the values of P@5, P@10, R@5 and R@10. When differences were found, a Bonferrone post-hoc test was applied to provide specific information on which data were different from each other. For all analyses, a significance value of p < 0.05 was adopted. All results are presented as the mean ± standard deviation.

## Results

Table 1 shows the results of agreement between the evaluators for the identification of the relevant images during the effectiveness evaluation process. It is possible to observe a moderate agreement between evaluators A x B and A x C regarding the identification of relevant images for queries in relation to the MFC descriptor. Among B X C, higher values were identified for Alldescriptors. Table 2 presents percentage results when the evaluators considered relevant (R) and non-relevant (NR) images.

**Table 1:** Results of the interpersonal agreement analysis to identify the relevant images for each descriptor among the 3 different evaluators (A, B, and C).

	A x B	A x C	B x C
Area/Perimeter	22.54	19.27	31.03
FA	35.57	24.24	21.50
Circularity	21.13	20.99	23.31
MF	14.64	07.40	16.57
Rectangularity	15.32	24.85	26.70
MFC	49.21	45.22	30.08
Alldescriptors	35.79	39.62	54.21

Abbreviations: FA = Fractal Area, MF = Maximum Fractal, MFC = Multiscale Fractal Curve.

**Table 2:** Results of the percentage of agreement between images considered not relevant (NR—NR) and images considered relevant (R—R) between 3 different evaluators.

	A x B		A x C		B x C	
	NR—NR	R—R	NR—NR	R—R	NR—NR	R—R
Area/Perimeter	76.66	4.66	80.00	3.33	79.66	5.00
FA	82.66	4.66	85.33	2.66	85.66	7.33
Circularity	78.33	4.00	74.66	5.00	77.00	4.66
MF	72.66	4.33	75.66	2.66	70.66	5.33
Rectangularity	77.66	3.33	79.00	4.33	76.66	5.33
MFC	52.00	24.00	57.33	17.00	45.66	21.00
Alldescriptors	50.00	20.33	54.00	19.33	51.00	27.33

Abbreviations: FA = Fractal Area, MF = Maximum Fractal, MFC = Multiscale Fractal Curve.

**Fig 3:**
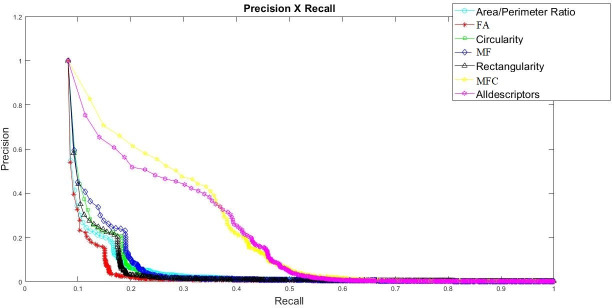
Representation of retrieved performance results in relation to the precision x recall curve for all evaluated descriptors. Abbreviations: FA = Fractal Area, MF = Maximum Fractal, MFC = Multiscale Fractal Curve.

Fig 3 illustrates the search performance, in terms of precision x recall, for the collection ranked in the seven evaluated descriptors. It can be seen that descriptors that obtain a greater number of information (feature vectors), such as the multiscale fractal curve (MFC) and then Alldescriptors (AllD), obtained better results of effectiveness, as confirmed in (Table 3). Statistical differences were observed among the descriptors for P@5 (*F* = 33.62; *p*< 0.001) and for P@10 (*F* = 32.15; *p*< 0.001). The post-hoc test showed for P@5 (*F* = 14.62; *p*< 0.001) that the results of the MFC and AllD descriptors presented statistically higher precision values in relation to all other descriptors. For P@10 (*F* = 11.76; *p*< 0.001), MFC and AllD also showed statistically higher precision values compared to all other descriptors. The descriptor FA showed statistical differences for MF in P@5 and P@10. For R@5, MFC and AllD presented statistically higher values in relation to all other descriptors (*F* = 21.45; *p*< 0.001). The descriptor FA showed lower values among MF. For R@10, MFC and AllD, the results were also greater in relation to all other descriptors (*F* = 22.26; *p*< 0.001).

**Table 3:** Values of mean ± standard deviation for precision (P@5 and P@10) and recall (R@5 and R@10) for evaluating the efficacy of different descriptors.

	Precision (P@5)	Recall (R@5)	Precision (P@10)	Recall (R@10)
Area/Perimeter	0.12±0.22	0.03±0.05	0.13±0.17	0.07±0.09
FA	0.09±0.13 ^†^	0.03±0.04 ^†^	0.09±0.13 ^†^	0.06±0.10
Circularity	0.14±0.18	0.04±0.08	0.14±0.16	0.09±0.10
MF	0.20±0.20	0.06±0.08	0.17±0.15	0.10±0.09
Rectangularity	0.14±0.17	0.04±0.06	0.14±0.14	0.09±0.09
MFC	0.46±0.37 ^#^*^×^^†^ª	0.14±0.13 ^#^*^×^^†^ª	0.40±0.31 ^#^*^×^^†^ª	0.24±0.19 ^#^*^×^^†^ª
Alldescriptors	0.43±0.36 ^#^*^×^^†^ª	0.13±0.11 ^#^*^×^^†^ª	0.39±0.32 ^#^*^×^^†^ª	0.24±0.20 ^#^*^×^^†^ª

Abbreviations: FA = Fractal Area, MF = Maximum Fractal, MFC = Multiscale Fractal Curve. The symbols indicate significantly differences (*p* < 0.05)

^#^different from area/perimeter ratio

*different from FA

^×^different from circularity

^†^different from MF

ªdifferent from rectangularity.

### Example of application using shape descriptors for match analysis in football

#### Data collect

For exemplification purposes, an application of the method was performed in a Brazilian professional football match, with application of the best evaluated descriptor. The 2D position of the players was obtained by a video-based system, and images of the shapes representing the organization of the teams were obtained (section “Elaboration of the polygons that represent the tactical organization of the football teams”) throughout the match for the first and second half of both teams (Team 1 and Team 2). Then, the MFC was calculated for the description of the images (section “Calculation of shape descriptors”)

#### Data analysis

With the MFC values for each image, k-means [34] was applied to cluster polygons to identify which shapes the teams adopted during the match. This clustering was performed using Euclidean distance (ED) data from the MFC feature vectors. The Elbow method [35] was used to identify the ideal number of k-clusters to be analysed, with the result of k = 8. After identifying which shapes were performed during the match, the number of shapes in each cluster was calculated as the percentage of occurrence in the match.

#### Results

**Fig 4:**
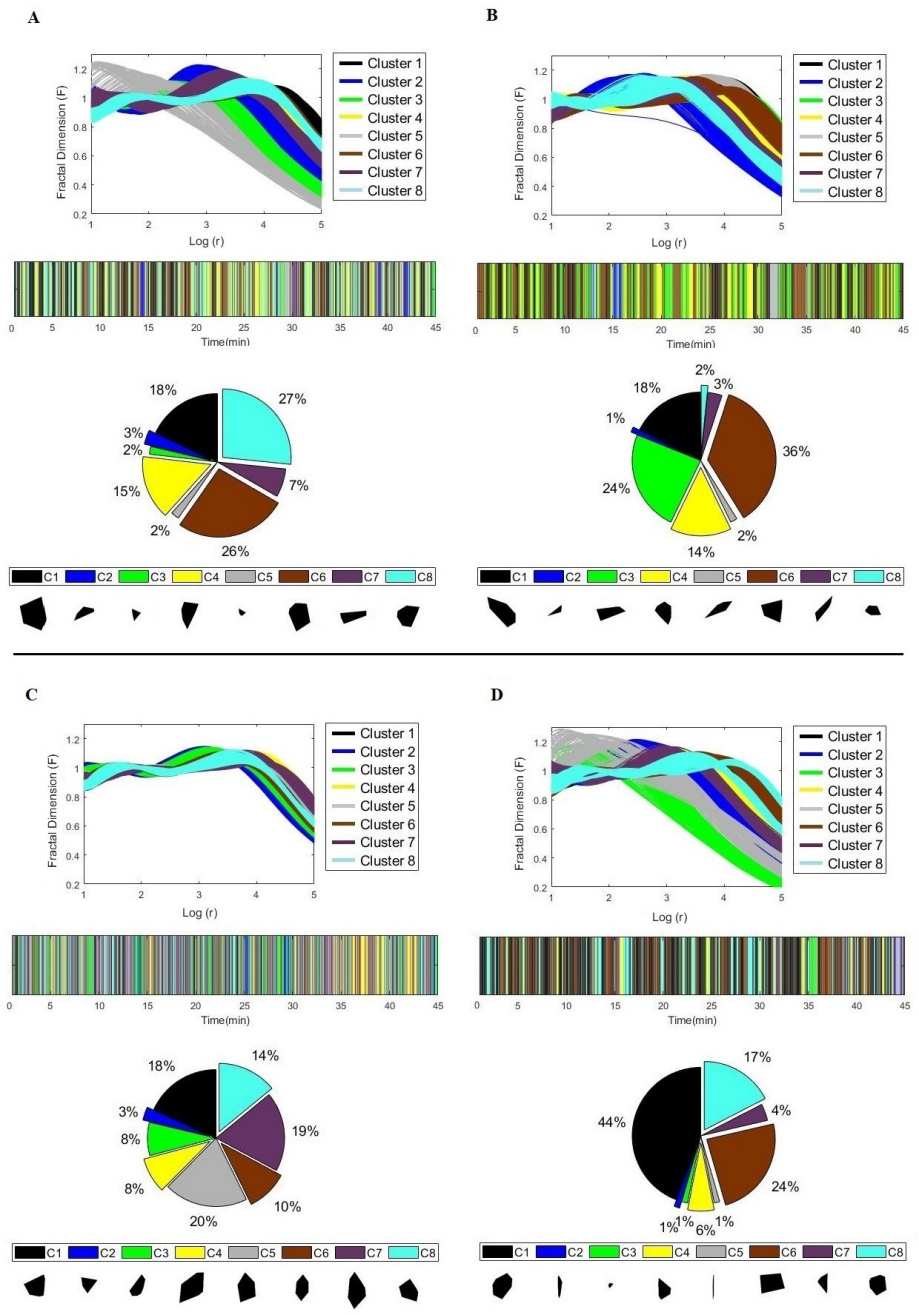
Result of clustering of MFC for Teams 1 and 2: (A) and (B), 1° and 2° half of Team 1, respectively; (C) and (D), 1° and 2° half of Team 2, respectively. Results too of the shapes found for each analysed cluster, when they occur throughout the match (bar graphic) and percentiles of shapes in each cluster (pie graphic).

Fig 4 shows the clustering results for the MFC of Team 1 (Fig 4A, 1° half; Fig 4B, 2° half) and Team 2 (Fig 4C, 1° half; Fig 4D, 2° half) in the 8 clusters. With the clustering of the MFC, it was possible to identify which shapes the teams perform during the match. Fig 4 too presents the results, with examples of which shapes can be observed during the match, when these shapes occur during the match (bar graphic) and the percentage of shapes (pie graphic) for each cluster (C1,. . ., C8) of Team 1 (Fig 4A, 1° half; Fig 4B, 2° half) and Team 2 (Fig 4C, 1° half; Fig 4D, 2° half).

## Discussion

The aim of this study was to evaluate different shape descriptors applied to images of polygons that represent the organization of football teams on the pitch and to establish which is the best descriptor to describe the shapes of team organization throughout a match. This study also presented an example of practical application of the best descriptor evaluated to identify the shapes that football teams are distributed on the pitch during the match.

Creating a content-based image retrieved system (CBIR) is not a simple task since it requires the creation of algorithms for encoding information from an image into feature vectors (FV) and similarity measures to compare a given image for collection images [28], which are commonly evaluated by real users [36, 37]. The results can be seen in (Fig 3), where it presents a result of the descriptor performance in relation to the precision x recall measures. We observed that the MFC descriptor presented a better performance for the retrieved images in relation to other descriptors evaluated, as well as the descriptor Alldescriptors presented a good performance. However, to obtain the values of the Alldescriptors of the images in the database, a higher computational cost was necessary to calculate all the descriptors and organize them in a FV, which makes the descriptor Alldescriptors less efficient. Even so, it is possible to highlight that descriptors with the greatest amount of information (feature vectors) presented better results, which can be confirmed in (Table 3). When observing the agreement results, it was possible to identify a moderate agreement to consider relevant images when they were retrieved using the MFC descriptor for two agreement analyses (A x B; A x C;—Table 1). When observing the percentage of agreement between the evaluators (A x B; A x C; B x C), especially between the relevant results (R–R) in the (Table 2), these values were low. However, it is possible to observe bigger percentage of agreement for the MFC descriptor, followed by Alldescriptors.

The results of this study corroborate the initial hypothesis that the shapes descriptor MFC may be the best one to describe the analysed phenomenon, since it presented better precision and recall values for P@5, R@5, P@10, and R@10 in relation to all descriptors evaluated, with the exception of the descriptor Alldescriptors. These results show that the MFC descriptor can be more robust in relation to the retrieval of images than other simpler descriptors, such as Area/Perimeter, Circularity and Rectangularity, which presented inferior results, which are invariant to scale, representing an important factor for the low performance of these descriptors.

The objects analysed in this study are images of the polygons formed by the convex hull of data from the position of football players. Football teams have different magnitudes of surface area in the context of the match, for example, when are with or without ball possession [3]. In this context, the teams may present different organization shapes during the match or even similar, but at different scales, which makes it a complex phenomenon for the detection of images, being necessary to perform an image transformation and detect these image variances [38]. Thus, a descriptor that considers the variation in scale may be the best option for this phenomenon, as was the case for MFC. This descriptor considers the representation of a shape for different scales; that is, each scale is related to different fractal dimension values [20]. Two other descriptors were also extracted from the MFC, such as FA and MF; however, a single value that describes the shapes did not prove to be good compared to a FV.

When considering the MFC as a good tool to describe the shapes of the organization of football teams, it was possible to apply it for the analysis in a football match of the Brazilian championship, allowing us to identify the most common shapes the teams performed during the match. Shapes with a larger scale were found more predominantly, such as (e.g. Fig 4D, C1, of Team 2). In a comparison between the curves, the shapes that are smaller (e.g. Fig 4A, C5, of Team 1), showed a decrease anticipatory in multiescale fractal dimension values in relation to the larger shapes (e.g. Fig 4A, C1, of Team 1). This happens due to the loss of format self-similarity in a given dilation when it increases or decreases [20].

In the literature, it is possible to observe studies that reported the control of space performed by teams on the pitch using the interpretation of the magnitude values of the surface area (represented by the hull convex), central aspect for the interpretation of these data [1, 3, 39]. When the values of the images of the hull convex are obtained and analyzed by the CFM values, it becomes possible to retrieve forms of organization that a given team performs. In the present study, we identified that both teams presented several different forms, on different scales, depending on different situations of the match. However, it was possible to verify that, even with the great variety of shapes that a given team can present, some of the shapes are consistent during the match. For example, team 1 presented the shape labeled “C6” during 36% of the second half (Fig 4B). Particularly, one can argue two important feedbacks to players and coaches with such analysis: a) the possibility of to confirm whether team organized the teammates accordantly to the strategy previously determined and b) the possibility of the coach to draw the team shape desired and then search in the data the moments that the team played accordantly.

Thus, by presenting an important tool such as the multiscale fractal dimension to describe the organization of football teams, it was possible to extract the shapes the football teams perform during a match. From these results, it is possible to think about applying this tool in important conditions during a match, when a team is in attack or defense (with and without ball possession) or analyses in determinant situations of a match, such as offensive sequences resulting in goals, or even to characterize the organizational shapes in the different systems of play (for instance, 4-4-2, 4-3-3, 3-5-2, etc…) adopted by teams of different countries and competitions.

## Limitations

It is also necessary to understand that the tool proposed is presented for a macro analysis perspective, considering the shape of the team formed by convex hull, neglecting, for example, the team players who are inside the polygon. Thus, future studies should be considered to improve this tool in more detailed analysis based on graphs, for example, considering all players on the pitch.

## Conclusion

The results allowed us to conclude that the multiscale fractal curve (MFC) descriptor is the most effective descriptor in relation to all the others evaluated in this study, which is important for describing shapes at different scales. Thus, it was possible to apply this descriptor during a Brazilian professional football match and identify the shapes the teams perform during the match. These results must be interpreted with caution, considering that it was applied only to one football match, for both teams. Therefore, the analysis should not be extrapolated to a whole context of the modality. Future studies should be applied with a greater number of matches so that we can characterize game patterns of different nationalities, as well as in different match contexts providing insights for coaches about the tactical performance of their teams or opponents.

## References

[1] Frencken W, Lemmink K, Delleman N, Visscher C. Oscillations of centroid position and surface area of soccer teams in small-sided games. European Journal of Sport Science. 2011;11(4):215–23. 10.1080/17461391.2010.499967. .

[2] Memmert D, Raabe D, Schwab S, Rein R. A tactical comparison of the 4-2-3-1 and 3-5-2 formation in soccer: A theory-oriented, experimental approach based on positional data in an 11 vs. 11 game set-up. PLOS ONE. 2019;14(1):1–12. 10.1371/journal.pone.0210191. PMC6353547 30699148

[3] Moura FA, Martins LEB, Anido RO, Barros RML, Cunha SA. Quantitative analysis of Brazilian football players’ organisation on the pitch. Sports Biomechanics. 2012;11(1):85–96. doi: 10.1080/14763141.2011.637123 22518947

[4] Sarmento H, Clemente FM, Araújo D, Davids K, McRobert A, Figueiredo A. What Performance Analysts Need to Know About Research Trends in Association Football (2012–2016): A Systematic Review. Sports Medicine. 2017;48(4):1–38. 10.1007/s40279-017-0836-6. 29243038

[5] Low B, Coutinho D, Gonçalves B, Rein R, Memmert D, Sampaio J. A Systematic Review of Collective Tactical Behaviours in Football Using Positional Data. Sports Medicine. 2020;50(2):1–43. doi: 10.1007/s40279-019-01194-7 31571155

[6] Rico-González M, Ortega JP, Nakamura FY, Moura FA, Arcos AL. Identification, Computational Examination, Critical Assessment and Future Considerations of Spatial Tactical Variables to Assess the Use of Space in Team Sports by Positional Data: A Systematic Review. Journal of Human Kinetics. 2021;77:205–21. doi: 10.2478/hukin-2021-0021 34168705 PMC8008301

[7] Rico-González M, Ortega JP, Nakamura FY, Moura FA, Los Arcos A. Origin and Modifications of the Geometrical Centre to Assess Team Behaviour in Team Sports: A Systematic Review. Revista Internacional de Ciencias del Deporte. 2020(b);16:318–29. 10.5232/ricyde2020.06106.

[8] Rico-González M, Ortega JP, Nakamura FY, Moura FA, Arcos AL. Identification, Computational Examination, Critical Assessment and Future Considerations of Distance Variables to Assess Collective Tactical Behaviour in Team Invasion Sports by Positional Data: A Systematic Review. International Journal of Environmental Research. 2020(a);17:1–14. doi: 10.3390/ijerph17061952 32192000 PMC7143020

[9] Bartlett R, Button C, Robins M, Dutt-Mazumder A, Kennedy G. Analysing Team Coordination Patterns from Player Movement Trajectories in Soccer: Methodological Considerations. International Journal of Performance Analysis in Sport. 2012;12(1):398–424. 10.1080/24748668.2012.11868607.

[10] Clemente M. F., Couceiro S. M., Martins F. M. L., Mendes R., Figueiredo AJ. Measuring Collective Behaviour in Football Teams: Inspecting the impact of each half of the match on ball possession. International Journal of Performance Analysis in Sport. 2017;13:678–89. 10.1080/24748668.2013.11868680.

[11] Duarte R., Araújo D., Folgado H., Eateves P., Marques P., Davids K. Capturing Complex, Non-Linear Team Behaviours During Competitive Football Performance. Journal of Systems Science and Complexity. 2013;26:62–72. 10.1007/s11424-013-2290-3.

[12] Frencken WGP, Lemmink KAPM. Team kinematics of small-sided soccer games. In: Reilly T, Korkusuz F, editors. Science and Football VI. New York: Routledge; 2009. p. 161–6.

[13] Vilar L, Araujo D, Davids K, Bar-Yam Y. Science of Winning Soccer: Emergent Pattrern-Forming Dynamics in association football. Journal of Systems Science and Complexity. 2013;26(1):1–13. 10.1007/s11424-013-2286-z.

[14] Locaric S. A survey of shape analysis techniques. Pattern Recogn. 1998;31(8):983–1001.

[15] Costa LF, Cesar RM Jr. Shape Classification: Theori and Practice. ed n, editor. Taylor & Francis Group 2009.

[16] Zhang DS, Lu GJ. Review of shape representation and description techniques. Pattern Recognition. 2004;37:1–19. 10.1016/j.patcog.2003.07.008.

[17] Mandelbrot BB. The fractal geometry of nature: W. H. Freeman and Co.; 1982.

[18] Chaudhuri B. B., Sarkar N. Texture Segmentation Using Fractal Dimension Ieee T Pattern Anal. 1995;17:72–7. 10.1109/34.368149.

[19] Fisher Y. Fractal image compression: theory and application. New York: Springer-Verlag. 1995.

[20] Torres RS, Falcão AX, Costa LF. A graph-based approach for multiscale shape analysis. Pattern Recognition. 2004;37:1163–74. 10.1016/j.patcog.2003.10.007.

[21] Figueroa PJ, Leite NJ, Barros RML. Background recovering in outdoor image sequences: An example of soccer players segmentation. Image and Vision Computing. 2006a;24(4):363–74. 10.1016/j.imavis.2005.12.012.

[22] Figueroa PJ, Leite NJ, Barros RML. Tracking soccer players aiming their kinematical motion analysis. Computer Vision and Image Understanding. 2006b;101(2):122–35. 10.1016/j.cviu.2005.07.006.

[23] Misuta MS. Analysis of the automatic tracking of players in collective sports. [Phd thesis]. Campinas: University of Campinas; 2009.

[24] Preparata FP, Shamos MI. Computational geometry: an introduction. New York: Springer-Verlag; 1985. xii, 390 p. p.

[25] Normant F, Tricot C. Method for evaluating the fractal dimension of curves using convex hulls. Physical Review A. 1991;43(12):6518–25. doi: 10.1103/physreva.43.6518 9905001

[26] Plotze RO, Falvo M, Pádua JG, Bernacci LC, Vieira MLC, Oliveira GCX, et al. Leaf shape analysis using the multiscale minkowski fractal dimension, a new morphometric method: a study with passiflora (passifloraceae). Canadian Journal of Botany. 2005;83: 287–301. 10.1139/b05-002.

[27] Sarkar N., Chaudhuri B. An efficient differential box-counting approach to compute fractal dimension of image. IEEE Transactions on Systems, Man and Cybernetics. 1994;24:115–20. 10.1109/21.259692.

[28] Torres RS, Falcão AX. Content-based image retrieval: Theory and applications. Revista de Informática Teórica e Aplicada. 2006;13(2):161–85.

[29] Penatti O. A. B., Valle E., Torres R. S. Comparative study of global color and texture descriptors for web image retrieva. Journal of Visual Communication and Image Representation. 2012;23:359–80. 10.1016/j.jvcir.2011.11.002.

[30] Gonçalves M. A., Fox E. A., Krowne A., Calado P., Laender A. H. F., Silva AS, et al. The effectiveness of automatically structured queries in digital libraries. ACM/IEEE Joint Conference on Digital Libraries. 2004:98–107. doi: 10.1109/JCDL.2004.1336106

[31] Baeza-Yates R., Ribeiro-Neto B. Modern Information Retrieval 1999.

[32] Cohen J. A coefficient of agreement for nominal scales. Educational and Psychological Measurement. 1960;XX(1):37–46.

[33] Landis JR, Koch GG. The measurement of observer agreement for categorical data. Biometrics. 1977;33(1):159–74. 843571

[34] Kaufman L, Rousseeuw PJ. Finding groups in data: an introduction to cluster analysis. New York: Wiley; 1990. xiv, 342 p. p.

[35] Han J, Kamber m, Pei j. Data Mining: Concepts and Techniques: Elsevier; 2012. 443–95 p.

[36] Abbasi S, Mokhtarian F, Kittler J. Enhancing CSS-based shape retrieval for objects with shallow concavities. Image and Vision Computing. 2000;18:199–211. 10.1016/S0262-8856(99)00019-0.

[37] Mokhtarian F, Abbasi S, Kittler J. Efficient and robust retrieval by shape content through cuvature scale space. Proceedings of the First International Workshop on Image Database and Multimedia Search. 1996:35–42.

[38] Aslandogan YA, Yu CT. Techniques and systems for image and video retrieval. IEEE Transactions on Knowledge and Data Engineering. 1999;11(1):56–63. doi: 10.1109/69.755615

[39] Rein R, Memmert D. Big data and tactical analysis in elite soccer: future challenges and opportunities for sports science. SpringerPlus. 2016;15(1410): 1–13. doi: 10.1186/s40064-016-3108-2 27610328 PMC4996805

